# Effects of a teacher-led social cognitive theory-based multicomponent movement education program on preschoolers’ fundamental movement skills and physical activity: the PA-REALITY cluster-randomized controlled trial

**DOI:** 10.1186/s12966-025-01864-y

**Published:** 2025-12-19

**Authors:** Amy S. Ha, Taoran Zeng, Meingold H. M. Chan, Cecilia H. S. Chan, Anthony D. Okely, Suzannie K. Y. Leung, Johan Y. Y. Ng

**Affiliations:** 1 Department of Health and Physical Education, The Education University of Hong Kong, Tai Po, New Territories, Hong Kong; 2 Department of Sports Science and Physical Education, The Chinese University of Hong Kong, Shatin, Hong Kong; 3 Department of Human Development and Family Science, Purdue University, West Lafayette, USA; 4 Early Start, School of Health and Society, University of Wollongong, Wollongong, Australia; 5 Department of Curriculum and Instruction, The Chinese University of Hong Kong, Shatin, Hong Kong

**Keywords:** Motor competence, TGMD-3, Physical activity, Head-toes-knees-shoulders task, Teacher professional development

## Abstract

**Background:**

A growing body of studies has shown that fundamental movement skills (FMS) and physical activity (PA) are crucial for preschoolers’ development. However, most Hong Kong preschoolers still do not meet the WHO guidelines for PA and demonstrate poor FMS performance. The present study examined the effectiveness of physical activity routines, education, assessment, literacy, and information technology application in young children (PA-REALITY), a social cognitive theory-based movement education program led by preschool teachers.

**Methods:**

Twenty-nine preschools signed up for the program and were cluster-randomized into an experimental group (15 preschools) and a wait-list control group (14 preschools). Totally 440 (age = 4 ± 0.95 years; 54.5% male) preschoolers took part in the baseline test and 349 preschoolers from 26 schools took part in the post-test (10 months). We delivered intervention contents including teacher workshops, teaching materials (booklet, simple sports equipment), and a mobile application to teachers. At baseline and post-test (10 months), respectively, preschoolers’ FMS proficiency, PA, and executive function (EF) were assessed using objective tools. Mixed-linear models using intention-to-treat analyses were used to examine intervention effects.

**Results:**

Participants in the experimental group showed significant improvements in catch (*B* = 0.37, *p* < .001) and moderate-to-vigorous PA (*B* = 4.49, *p* = .04) at 10 months, compared with participants in the wait-list control group. No effects were found for total PA other FMS and EF outcomes.

**Conclusions:**

The PA-REALITY intervention improved some FMS outcomes and MVPA at 10 months. These results highlight the important roles of teachers in developing children’s movement skills and PA. Continuous professional development training for teachers may be an effective and sustainable way to improve existing practices.

**Supplementary Information:**

The online version contains supplementary material available at 10.1186/s12966-025-01864-y.

## Introduction

Regular physical activity (PA) promotes growth and development and has multiple benefits for physical, mental, and psychosocial health of children [1]. Developing PA habits during the early years may have long-lasting benefits throughout an individual’s life course, such as adiposity [2], bone and skeletal health [2], motor skill development [3], psychosocial health [4], cognitive development [5], and cardiometabolic health [2, 6]. For preschoolers, the World Health Organization (WHO) [7] recommends engagement in a range of PA of varying intensities (from light to vigorous intensities) for 180 min every day. However, a few preschoolers in Hong Kong meet these standards, which is worrisome [8, 9]. A key contributing factor to young children’s PA is their proficiency in fundamental movement skills (FMS), including locomotor (e.g. running and jumping), object control (e.g. throwing and kicking), and stability (e.g. balancing). FMS are considered the prerequisites or “building blocks” of many physical activities and sports [10]. In addition to supporting an active lifestyle, researchers have shown that proficiencies in these skills are associated with better cardiovascular fitness and healthier weight statuses [11]. Therefore, building children’s FMS proficiency from a young age is important [12].

Intervention is required to advance knowledge of both teachers and parents of young children, in terms of how to improve or sustain both the quantity and quality of movement behaviours in young children. More broadly, there is a need to promote physical literacy [13], a broad concept that encompass the motivation, confidence, knowledge, value, and behaviours pertaining PA, amongst preschoolers, their teachers, and their parents.

Despite a growing awareness, Hong Kong preschoolers face many challenges regarding their physical developments. For instance, academic achievements of children are valued to a larger degree than other aspects of development in Chinese societies [14]. As such, many parents devote significant time and resources to support their children’s cognitive development compared to other aspects. Moreover, limited recreational space and facilities discourage physical activity for both parents and children [15]. In fact, many parents in Hong Kong perceive high barriers to their own physical activities [16], such as a lack of time, motivation, facilities, and childcare responsibilities. These barriers making it even more challenging to find time to be active with their children. Unfortunately, these challenges were accentuated during the COVID-19 pandemic, with many children being restricted to their own homes [17]. Similar barriers can also be found in school settings as preschoolers may be constrained to limited types of activities due to a lack of space in school environments [18, 19]. Further, teachers in preschools may face additional challenges [19]. First, there are no structured physical education sessions or curricula in Hong Kong preschools. Instead, daily PA sessions, usually taught by generalist teachers, support the development of children’s FMS. Yet most preschool teachers are trained as generalists, hence few have received structured, professional training in physical education or FMS teaching. As such, the quality of their instruction may be suboptimal [19]. Results from a meta-analysis suggested that the delivery of FMS instruction by highly trained physical education teachers would improve students’ proficiency in these skills [20]. Accurate assessment and feedback provision are also important in quality education. Due to insufficient training in these areas, preschool teachers may be undertrained in assessing children’s performances and providing appropriate feedback to help students improve. As teachers are the backbone in preschool education, therefore, continuing professional development for teachers would benefit both teachers and children [21].

In this study, we evaluated the physical activity routines, education, assessment, literacy, and information technology application in young children (PA REALITY) program, which comprised intervention components such as teacher professional development training, teaching materials and simple sports equipment to schools and families, and access to a mobile application. A cluster-randomized controlled trial was conducted to evaluate the effectiveness of the intervention. We hypothesised that children who received the intervention, compared to those who did not, would demonstrate larger improvements in terms of the measured outcomes after receiving the intervention.

## Method

### Trial design

To evaluate the effectiveness of PA-REALITY program intervention components, a two-arm cluster-randomized controlled trial was conducted. Preschools were randomly allocated (1:1 ratio) to an experimental group or a wait-list control group (who will receive the intervention 11 months later). Changes in the primary outcomes of PA behaviours (measured with accelerometery) and FMS from pre- to post-intervention were compared. The current study examined the intervention effectiveness with the data at baseline and post-test (10 months). Ethical approval was obtained from the Joint Chinese University of Hong Kong – New Territories East Cluster Clinical Research Ethics Committee (ref: 2022.078). This trial was registered with the National Clinical Trials Registry, number NCT06742294.

### Participants

Preschoolers aged 3–5 with no history of neurological, psychiatric or physical illnesses were eligible to participate. We emailed and posted the project recruitment leaflet to 1,030 local preschools in Hong Kong between May 2022 and June 2023 (CONSORT checklist in supplementary material). Eighty-nine preschools signed up for the project briefing sessions and 29 of them eventually agreed to participate. Participants were recruited through schools, with 15 preschoolers randomly selected from each (except for one school in the experimental group, which had 20 children), five in each grade level, i.e., K1 (age 3–4); K2 (age 4–5); K3 (age 5–6). Therefore, a total of 440 preschoolers were assessed with their parents’ written consent. At the post-test (10 months), three schools (two in experimental group) dropped out and 349 preschoolers (90%) took part in the post-test.

### Interventions

Social cognitive theory (SCT) [22] posits that personal factors, the social environment, and behaviours are intricately and reciprocally related. Behaviours of preschoolers, by and large, are shaped by key socialising agents (i.e., parents and teachers) and the environments created by these key members. Therefore, SCT is an appropriate framework for designing PA interventions. As such, we developed the PA-REALITY program, which commenced in May 2022, to improve the quantity and quality of PA in preschoolers. Specifically, intervention components were designed to target six key SCT constructs, namely self-efficacy, behavioural capacity, outcome expectations, self-regulation, social environment, and physical environment (For details, see supplementary material Table 1). We developed and delivered invention elements including teaching materials, face-to-face and online workshops, coach demonstration, and a smartphone-based FMS-rating system to teachers to enhance their physical literacy, self-efficacy and professional development (see supplementary material for details). Since children spend much time at home, a parental booklet and a set of simple sports equipment were developed for parents to teach and co-engagement in PA with preschoolers at home to strengthen the intervention effect. We aimed to improve preschoolers’ FMS performance and PA by enhancing the social and physical environment at school and at home via the common effort of teachers and parents (i.e., home-school cooperation).

### Outcomes

The primary outcomes of the randomized controlled trial were children’s FMS proficiency and PA. The indicators for PA included MVPA and total PA because they were referenced in the WHO’s (2019) movement behaviour guidelines for young children. Their executive function was measured and used as a secondary outcome. Children’s FMS and EF were assessed at their preschools by the research team. Accelerometers were administered to participants either before or immediately after they completed the FMS and EF assessments.

#### Fundamental movement skill proficiency

Fundamental movement skill proficiency was measured using the Test of Gross Motor Development-3rd Edition (TGMD-3) protocols [23]. Specifically, all participants in different grades were tested on four skills (jump, single-leg balance, kick, and overhead throw), which are crucial for preschoolers to learn more complicated movements or sports [10]. Catch and hop were additional assessments for children in the 2nd and 3rd grades (K2 and K3), while skip was an additional assessment for K3 children only. Prior to the evaluation, preschoolers were arranged into groups of three to five, and a trained research assistant showed the children a standardized video demonstrating each skill to ensure accurate execution. Each skill was evaluated twice on three to five performance criteria. The children completed these assessments individually in about 15 min and their performance was video-recorded. All participants’ FMS performances were then rated by the same research assistant, who was trained to rate FMS videos according to TGMD-3. According to the TGMD-3 protocol, catch and skip were evaluated against three performance criteria while jump, hop, kick and overhead throw were evaluated against four criteria. For each criterion, and trial, children’s performance was marked either one or zero, where the quality of action is performed or not presented, respectively. As a result, the maximum point for catch and skip was three, for jump, hop, kick and overhead throw was four, and for single-leg balance was five. Based on Movement Assessment Battery for Children-2 (M-ABC) [24], single-leg balance was evaluated, with the maximum time of 30 s each trial.

#### Physical activity

Participants were instructed to wear an ActiGraph wGT3X-BT accelerometer (ActiGraphTM, Pensacola, FL) on an elastic waistband around their waist for five consecutive days, except during water-based activities such as showering or swimming. Data were recorded in 15-second epochs and considered valid if participants wore the devices for eight hours or more on at least two weekdays and one weekend day. Time spent sedentary (≤ 799 counts/15-sec), in light-intensity PA (LPA; 800–1679 counts/15-sec), moderate intensity PA (1680–3367 counts/15-sec), and vigorous intensity PA (≥ 3368 counts/15-sec) during waking hours were defined and calculated using the cut-points developed by Pate and colleagues [25]. These cut-points were chosen as they were also adopted by an international surveillance study of young children’s movement behaviours [26]. Using the same cut-points would enable meaningful comparisons between our results and those from related studies conducted globally. The average times (across all valid days) spent in LPA and moderate-to-vigorous physical activity (MVPA, the sum of moderate and vigorous intensity PA) were used as the outcome for analyses.

#### Executive function

Executive function was assessed using the Head-Toes-Knees-Shoulders-Revised task(HTKS-R) [27]. HTKS was first developed by Ponitz and colleagues [28] as a direct assessment of inhibitory control (a child must inhibit the dominant response of imitating the examiner), working memory (a child must remember the rules of the task) and attention focusing (a child must focus attention to the directions being presented by the examiner). It is a more interactive, toolless, inexpensive and feasible assessment [27, 29] compared with computer-based tools. It also more consistently predicts math and literacy than individual EF measures assessing inhibitory control, working memory, and task shifting [30]. We applied the revised version HTKS-R, which reduces the floor effect [27], in the current study. A total of 59 items (22 practice items and 37 test items) across four parts (Parts 0–3) instructed children to say or do the opposites of command(s). In the initial part, children were asked to say/touch their heads, toes, shoulders or knees, in which children should respond as instructed. In the following stages, children should respond in the “opposite” way (e.g., touch their heads when the examiner said “touch your toes”). The tests took 5 min per child on average to complete. For each item, incorrect responses were scored as 0, self-corrected responses were scored as 1, and correct responses were scored as 2. Total scores range from 0 to 118, higher score indicating better executive function. The HTKS-R has demonstrated strong internal consistency in diverse samples around the world [27]. The reliability in our study measured with Cronbach’s α of 59 items was 0.98. To mitigate the tester bias, one research assistant administered this test to all participants.

#### Demographic information

Children’s gender, age, their parents’ educational level and employment status, monthly household income, and number of children were collected via a parent-report questionnaire.

### Sample size calculation

The required sample size was calculated based on a prudent expectation to achieve a small effect (Cohen’s d = 0.2) in terms of primary outcome of accelerometer-measured physical activity in preschoolers. Using G*Power 3.1.9.4 with a 1:1 experimental versus control ratio, an alpha level at 0.05 and a power of 0.8, the required total sample size will be 156. To avoid potential contamination of intervention effects within schools, randomization was conducted by clusters, at the school level. Based on previous work conducted in Hong Kong, the compliance rate of deployed accelerometers is approximately at 70% [8]. Based on this rate, and from a cluster size of 15 (i.e., 5 children per grade), the effective average cluster size is 10.5. With an intraclass coefficient of 0.07 [8], the design effect is 1+(10.5-1) * 0.07 = 1.67. Therefore, the sample size required is 260, and therefore participants from 26 schools were required in the main trial. To account for potential dropouts, we recruited additional schools to take part.

#### Randomization and allocation concealment

**Fig. 1:**
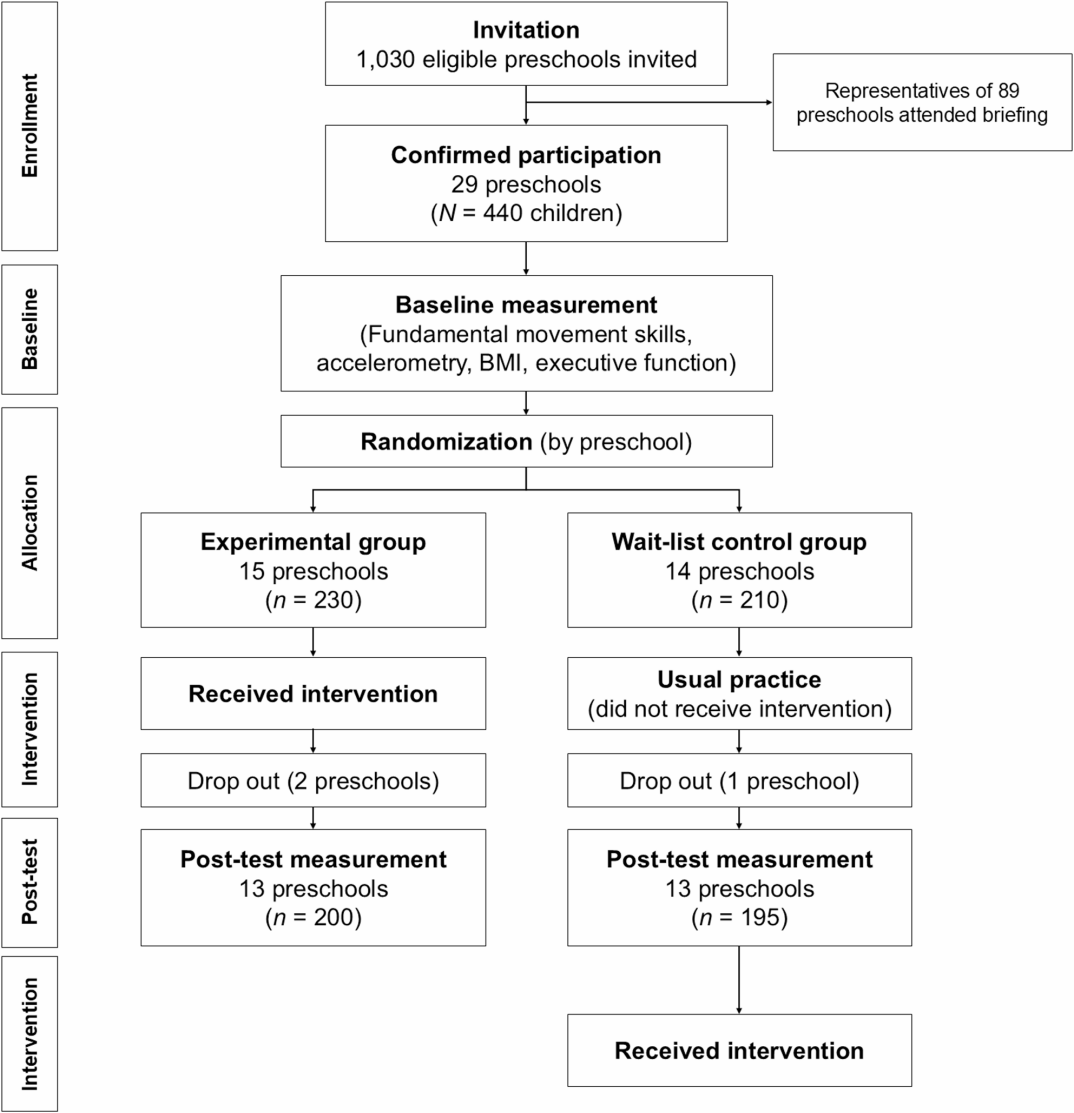
Flow diagram of study

Randomization was done (by JN) using a computer-based random number generator, after data collection at baseline was completed. The schools were the unit of randomization. Using this randomization sequence, half of the schools were allocated to the experimental group, and the other schools to the control group. As teachers at schools allocated to the experimental group would receive professional development training, allocation concealment or blinding was not possible (Fig. 1).

### Data analyses

Intervention effects were analysed using an intention-to-treat principle, meaning that all children with data were included irrespective of them receiving the intervention or not [31]. Also, when participants had missing data at post-intervention, the corresponding values at baseline were imputed as the post-intervention value prior to data analyses. Mixed linear models were used to account for the clustering of children within preschools. This method is suitable for longitudinal data and handles missing data well [32]. The analyses compared pre-to-post intervention changes between experimental and control groups. Effect estimates were derived from testing the interaction group*time for post-test (B3*Time*Group) in Eq. 1.

1 $$\mathrm{Outcome}=\mathrm{B}0+\mathrm{B}1*\mathrm{Time}+\mathrm{B}2*\mathrm{Group}+\mathrm{B}3*\mathrm{Time}*\mathrm{Group}$$

Effect estimates were reported as regression coefficients (*B*), with 95% confidence intervals (CI), *p*-values, and ICCs for the clustering effect at school level, and Cohen’s *d* (comparing change scores). Children’s age at baseline was included in the models as a covariate. We specified a random intercept and a fixed slope to characterize changes over time (the data did not support random slopes) [5, 33]. All mixed linear models were conducted using the *lmer* package [34] in RStudio v. 4.4.2.

## Results

### Randomization allocation and final sample

Using our randomization sequence, 230 children from 15 preschools were allocated to the experimental group, and 210 children from 14 preschools were in the wait-list control group. Participants who had valid data on at least one of the outcomes at baseline were included in our analyses. Children’s demographic background and baseline characteristics are shown in Table 1. Table 2 shows baseline and post-test levels for primary and secondary outcomes. The results from the independent sample t-tests or Chi-squared tests indicated that there were no baseline differences between the experimental and control groups in all variables (*p*s > 0.05). At post-test, outcomes for 91 participants (57% from the experimental group) were missing due to children dropping out of school or being absent, and these were imputed using their corresponding baseline scores. Analyses were conducted based on intention-to-treat principles, with all participants being included in their original assigned groups.

**Table 1:** Demographic background of participating children

	Experimental group	Wait-list control group
	Mean (SD) or percentage	
Age (years)	4.0 (0.95)	4.0 (0.95)
Gender (% boys)	55.5	53.6
BMI(kg/m²)	15.7 (2.9)	15.7 (1.6)
Father’s age (%)		
≤ 29 years	2	3
30–34 years	12	12
35–39 years	27	20
40–44 years	23	22
≥ 45 years	11	7
Did not report	25	36
Mother’s age (%)		
≤ 29 years	6	6
30–34 years	16	22
35–39 years	33	26
40–44 years	19	14
≥ 45 years	4	1
Did not report	22	31
Father’s highest education level (%)		
Primary school	1	2
Secondary school	35	39
Associate degree or higher diploma	12	7
Bachelor’s degree	21	14
Master’s or doctorate degree	5	4
Did not report	26	34
Mother’s highest education level (%)		
Primary school	1	3
Secondary school	36	36
Associate degree or higher diploma	19	16
Bachelor’s degree	18	12
Master’s or doctorate degree	3	3
Did not report	23	30
Father’s employment status (%)		
Househusband	2	0
Full-time	66	60
Part-time	4	4
Unemployed	0	2
Retired	1	0
Did not report	27	34
Mother’s employment status (%)		
Housewife	34	33
Full-time	32	24
Part-time	12	12
Unemployed	0.4	0
Retired	0	0
Did not report	21.6	31
Marital status (%)		
Married	70	62
Single	3	1
Divorced	4	6
Widow	0	0
Other	0.4	1
Did not report	22.6	30
Household monthly income (%)		
HK$≤ 9,999	2	2
HK$10,000–19,999	10	15
HK$20,000–29,999	16	17
HK$30,000–39,999	15	13
HK$40,000–59,999	15	11
HK$60,000–79,999	9	4
HK$80,000–99,999	4	3
HK$≥100,000	5	5
Did not report	24	30

**Table 2:** Baseline and post-test levels of mean (SD) for primary and secondary outcomes

Outcomes	Experimental group		Wait-list control group	
	Pre	Post	Pre	Post
MVPA(min/day)	67.37 (25.86)	67.39 (24.50)	63.93 (35.03)	59.46 (28.17)
TotalPA(min/day)	145.18 (44.56)	144.78 (44.63)	137.04 (70.75)	132.75 (55.52)
Catch	1.51 (0.71)	1.86 (0.70)	1.60 (0.89)	1.58 (0.72)
Jump	0.94 (0.83)	1.34 (1.07)	0.98 (0.84)	1.24 (0.96)
Overhead throw	0.44 (0.71)	0.81 (0.99)	0.40 (0.68)	0.65 (0.92)
Kick	2.37 (1.03)	2.56 (0.93)	2.34 (1.19)	2.41 (0.98)
Hop	1.22 (0.86)	1.39 (0.74)	1.31(0.82)	1.39 (0.82)
Skip	0.79 (0.74)	0.93 (0.78)	0.69 (0.35)	0.79 (0.81)
Balance (s)	3.09 (1.45)	3.47 (1.48)	2.87 (1.73)	3.17 (1.64)
EF	43.19 (36.48)	65.78 (37.00)	41.72 (39.68)	62.09 (41.94)

*TotalPA *light and moderate to vigorous physical activity, *MVPA* moderate to vigorous physicalactivity,*EF* executive function.

### Fundamental movement skill proficiency

Our results suggested that children’s proficiency in catch did not change over time (*B* = -0.02, 95% CI [-0.16, 0.12], *p* = .75), and there were no main effects for group allocation (*B* = -0.08, 95% CI [-0.26, 0.09], *p* = .37). However, there was a significant intervention effect (*B* = 0.37, 95% CI [0.18, 0.55], *p* < .001) in favor of the experimental group.

Similar results were found for the skills of jump, overhead throw, and single-leg balance. For overhead throw, children’s scores improved over time (*B* = 0.23, 95% CI [0.11, 0.35], *p* < .001), but the effects for group (*B* = 0.05, 95% CI [-0.14, 0.24], *p* = .60) or intervention (*B* = 0.06, 95% CI [-0.10, 0.22], *p* = .49) were not significant. With regards to jump, a significant time effect was found (*B* = 0.26, 95% CI [0.12, 0.40], *p* < .001), with no group (*B* = -0.03, 95% CI [-0.23, 0.17], *p* = .79) or intervention (*B* = 0.14, 95% CI [-0.05, 0.33], *p* = .16) effects. For single-leg balance, children’s scores also improved over time (*B* = 4.49, 95% CI [3.43, 5.55], *p* = < 0.001), but the terms for group allocation (*B* = 1.83, 95% CI [-0.13, 3.80], *p* = .08) and the interaction term (*B* = -1.22, 95% CI [-2.68, 0.25], *p* = .11) were not.

For the remaining FMS skills of kick, hop, and skip, all the examined terms in the models were not significant. Detailed results of these analyses are shown in Table 3.

**Table 3:** Results of main analyses

Outcome	*n*	Max score	ICC	Time		Group		Time * Group	
				B, 95% CI	*p*	B, 95% CI	*p*	B, 95% CI	*p*
FMS									
Catch	298	3	0.03	-0.02 [-0.16, 0.12]	0.75	-0.08 [-0.26, 0.09]	0.37	0.37 [0.18, 0.55]	< 0.001
Jump	432	4	0.03	0.26 [0.12, 0.40]	< 0.001	-0.03 [-0.23, 0.17]	0.79	0.14 [-0.05, 0.33]	0.16
Overhead throw	440	4	0.05	0.23 [0.11, 0.35]	< 0.001	0.05 [-0.14, 0.24]	0.60	0.06 [-0.10, 0.22]	0.49
Balance	434	5	0.04	4.49 [3.43, 5.55]	< 0.001	1.83 [-0.13, 3.80]	0.08	-1.22 [-2.68, 0.25]	0.11
Kick	439	4	0.03	0.06 [-0.10, 0.19]	0.36	0.04 [-0.19, 0.25]	0.72	0.12 [-0.04, 0.29]	0.20
Hop	296	4	0.03	0.08 [-0.06, 0.23]	0.27	-0.08 [-0.28, 0.13]	0.48	0.09 [-0.11, 0.31]	0.36
Skip	154	3	0.09	0.10 [-0.06, 0.27]	0.21	0.11 [-0.18, 0.40]	0.45	0.04 [-0.18, 0.26]	0.73
Physical activity									
MVPA	286	-	0.17	-4.47 [-7.47, -1.46]	0.004	3.67 [-6.09, 13.42]	0.47	4.49 [0.32, 8.65]	0.04
Total PA	286	-	0.27	-4.29 [-9.52, 0.94]	0.11	10.79 [-13.25, 28.92]	0.47	3.89 [-3.37, 11.16]	0.29
Executive function	440	60	0.08	15.04 [11.58, 18.50]	< 0.001	1.81 [-6.59, 10.23]	0.68	3.76 [-0.99, 8.52]	0.12

### Physical activity

We conducted analyses with respect to children’s MVPA and total PA (i.e., combined light, moderate, and vigorous forms of activity). Our results suggested that children’s MVPA decreased over time (*B* = -4.47, 95% CI [-7.47, -1.46], *p* = .004). There were no group differences (*B* = 3.67, 95% CI [-6.09, 13.42], *p* = .47), but an intervention effect (*B* = 4.49, 95% CI [0.32, 8.65], *p* = .04) in favour of the experimental group was found. By contrast, our results with respect to total PA showed there were no time (*B* = -4.29, 95% CI [-9.52, 0.94], *p* = .11), group (*B* = 7.85, 95% CI [-13.25, 28.92], *p* = .47), or intervention effect (*B* = 3.89, 95% CI [-3.37, 11.16], *p* = .29).

### Executive function

Analyses were conducted for children’s EF, as a secondary outcome of the trial. Results suggested that children’s scores improved significantly over time (*B* = 15.04, 95% CI [11.58, 18.50], *p* < .001). However, the difference between groups (*B* = 1.81, 95% CI [-6.59, 10.23], *p* = .68) was not significant, and there were no intervention effects (*B* = 3.76, 95% CI [-0.99, 8.52], *p* = .12).

## Discussion

The PA-REALITY intervention was designed to improve preschoolers’ fundamental movement skills and physical activity through a movement education program delivered by trained, in-service teachers. Grounded in SCT, the program was designed to first enhance teachers’ knowledge, pedagogical skills, and assessment literacy related to physical literacy and FMS instruction, thereby improving teachers’ classroom instructional practices and ultimately generating a positive impact on children’s FMS and PA performance. Despite a relatively indirect pathway, our findings suggest the intervention was successful in improving children’s proficiency in catch and MVPA at 10 months. These results align with those from meta-analysis [35] and other interventions among preschoolers, where intervention effects were mostly found in ball skills, but not locomotor skills [36–38]. Nevertheless, there were several key differences between our program and those in previous studies. For example, instead of relying on external sources or providers, we aimed to empower in-service teachers with knowledge and skills to eventually deliver the intervention contents. In our post-test semi-structured interviews with participated teachers, most of them reported that the trainings improved their physical literacy and teaching skills, and that they have continued to use the teaching materials in physical education since the intervention. While indirect, this mode of delivery is potentially more scalable and sustainable, and may be more advantageous compared to previous approaches [39]. Interventions using similar teacher-training has been found effective in improving preschoolers’ PA performance and health [40, 41]. Second, previous intervention programs for preschoolers were primarily designed to improve children’s PA, but they did not directly manipulate children’s motor skills [42]. However, research suggested that children with low FMS capability also showed low motivation for PA [43, 44]. Therefore, including direct intervention strategies targeting FMS is pivotal and was employed in the current program.

Our results also provide some insight into the status quo of motor skill instruction in Hong Kong preschools. For instance, our results suggested that participants did not show improvements over time for four of the FMS we assessed (i.e., catch, kick, hop, and skip). This lack of improvement may be due to an absence of teaching these skills, or that the corresponding instruction was subpar. Either explanation suggests that insufficient importance is placed on children’s motor skill development, which further underscores the significance of our approach in enhancing teachers’ professional skills and knowledge. From a more macro perspective, this also points towards a need for stronger support in terms of curricula or school policy [45]. In terms of children’s MVPA, we found that children had an overall reduction in MVPA levels, which was similar to previous studies [46, 47], but our intervention effectively negated this downward trend in the intervention group. As MVPA has been shown to be associated with many beneficial health outcomes [1, 2], this negative trend is concerning. Regular monitoring of children’s activity levels may be needed to raise awareness and reduce the decline in their PA. While we found that the intervention improved children’s MVPA, similar improvements were not observed for total PA. One possible explanation is that children’s time allocation for sleep, meal, school, and other activities is, by and large, structured by preschools and parents such that they may not offer great flexibility for change. Allocating additional time for PA, both in-school and out-of-school, may be challenging. This may explain why the intervention was unsuccessful in increasing overall PA time [48]. Nonetheless, with increased knowledge and awareness, teachers and parents may be able to provide opportunities for their children to perform PA at a higher intensity within the available time. This explanation would yield results consistent to our findings, and would underscore the positive effects of our intervention.

While the PA-REALITY intervention has shown some improvement in children’s outcomes, several factors might have limited its potential impact. First, the level of engagement among preschools that took part in the current trial varied greatly. While the actual level of commitment may be difficult to quantify, attendance numbers to teacher workshops would suggest that certain preschools only devoted minimal resources to the project (with only one staff appointed to attend), while some other schools had a much stronger attendance. To minimize the impact of teachers’ absence from workshops, we provided video recordings of each session for their review and discussion. Nevertheless, the commitment of schools directly affects how much the intervention’s content and objectives are integrated into teachers’ instruction, and in turn impacts children’s learnings [49]. Additionally, the availability of space within school premises may also affect teachers’ instruction. In the current study, some preschools had very limited physical space for activity sessions, which posed challenges to intervention delivery [18]. One component of the current intervention was to provide teachers and parents to a mobile application that could be used to assess children’s motor skills. Unfortunately, the application’s usage rates were low, which may have limited intervention effectiveness. However, researchers have found that eHealth approaches can be effective in improving children’s PA [50, 51]. Electronic communication channels could be better utilized to deliver intervention-related knowledge, information, or reminders to participants to enhance program effects [52]. Additionally, since children spend substantial time at home with their parents, the positive effects of the intervention would likely be greater if parents actively participated, such as by practicing FMS or engaging in PA together with their children [53].

### Strengths and limitations

The PA-REALITY intervention has several strengths. First, we employed a sustainable and scalable intervention approach by equipping teachers with professional knowledge and skills in physical education, instead of inviting external coaches or providers to deliver the intervention. Coach-led or researcher-led physical activity interventions for preschoolers may lack sustainability once external experts are no longer involved and lack first-hand/insider knowledge about school settings and children [54]. Second, the teaching focus on FMS included not only deliberate instruction but also intentional play through designed games that incorporated motor skills, which contributed to the intervention’s effectiveness [35]. There are also several limitations to the study. For example, during the intervention phase, teachers’ instructions were not closely monitored by the research team. This was due to challenges to logistical arrangements, and teachers’ concerns over privacy issues related to video-recording their classes. As a result, we had limited information in terms of the fidelity of intervention delivery. Nevertheless, class observations were arranged for all preschools during the intervention delivery phase, and researchers provided immediate feedback on teachers’ strengths and weaknesses in their instruction, and also engaged in an extensive discussion on future improvements. These measures should ensure classes to be delivered at a sufficient level of standard.

## Conclusion

Based on SCT, the PA-REALITY intervention was designed to improve preschoolers’ PA and motor skills via improving their teachers’ physical literacy and preschools’ teaching environment. Results from our randomized controlled trial provide preliminary evidence of teacher-training efficacy that preschoolers’ FMS and PA levels were partly improved though they were not directly trained but their teachers were. While such success in a teacher-led intervention is encouraging, our results highlight the need for more systemic support and enhancements within preschool curricula. Regular monitoring of children’s physical activity and motor skills will be beneficial, and could be achieved using digital tools. Future studies should investigate the views of teachers and school administrators on similar initiatives, and in turn design more effective ways to scale up the program. Ultimately, parents also play an important role in children’s development and facilitating home school cooperation may maximize intervention potentials.

## Supplementary Information

Supplementary Material 1.

Supplementary Material 2.

Supplementary Material 3.

## Data Availability

﻿** The datasets used and/or analysed during the current study are available from the corresponding author on reasonable request.

## Abbreviations

CI: Confident interval
EF: Executive function
ES: Effect size
FMS: Fundamental motor skills
LPA: Light physical activity
sec: Seconds
min: Minutes
MVPA: Moderate-to-vigorous physical activity
PA: Physical activity
SCT: Social cognitive theory
SD: Standard deviation
TotalPA: Light and moderate to vigorous physical activity
WHO: World Health Organization
