# Supplementary material for: Effects of a teacher-led social cognitive theory-based multicomponent movement education program on preschoolers’ fundamental movement skills and physical activity: the PA-REALITY cluster-randomized controlled trial
Source: Int J Behav Nutr Phys Act. 2025 Dec 19;23:8. doi: 10.1186/s12966-025-01864-y (PMC12837030; doi:10.1186/s12966-025-01864-y)
Supplement: Supplementary file 3 — Supplementary Material 3. [file 12966_2025_1864_MOESM3_ESM.docx]

Materials and Procedures of the PA-REALITY intervention


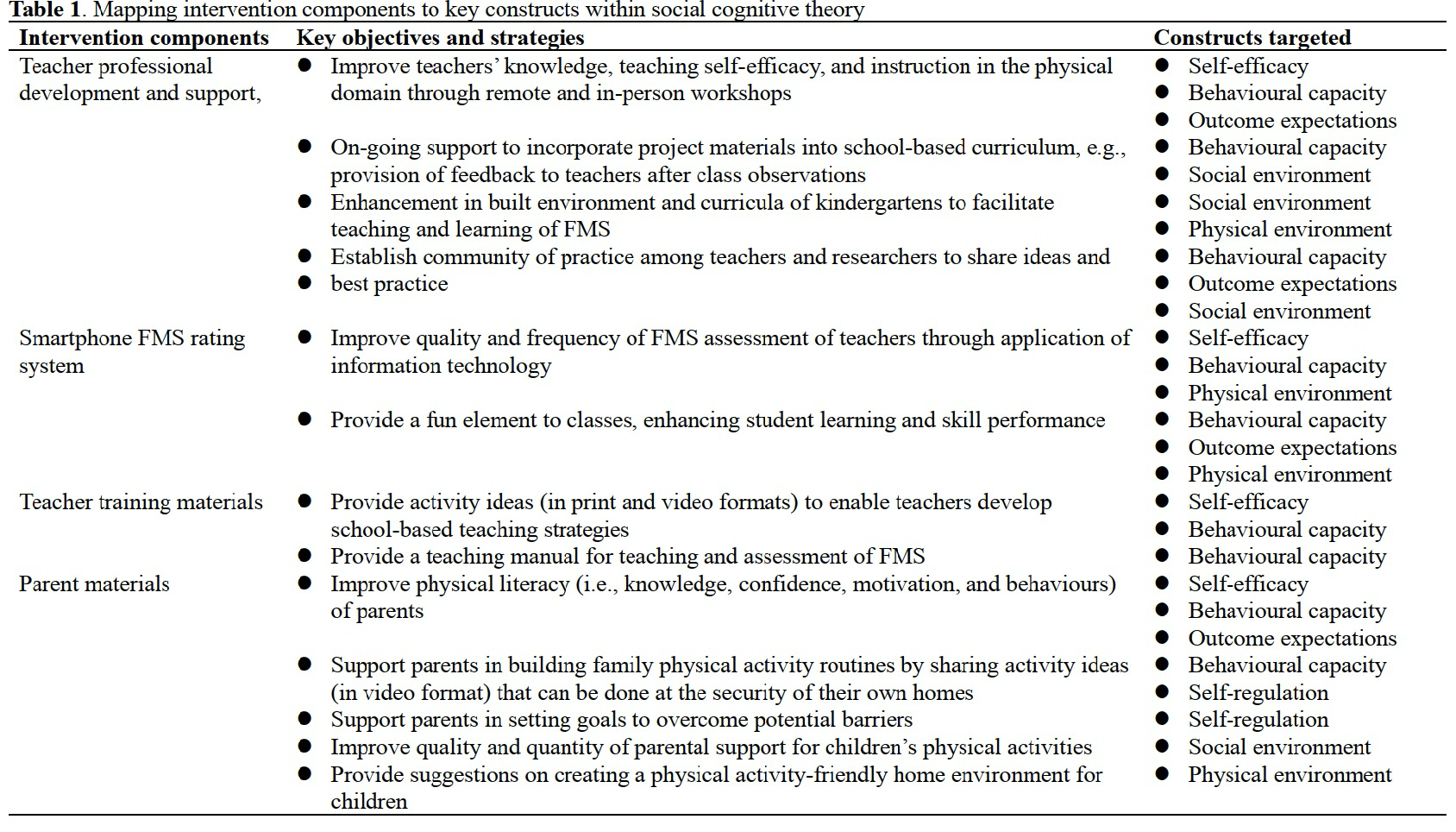


Table 2 Intervention details

| Month | 1st | 2nd | 3rd | 4th | 5th | 6th | 7th | 8th | 9th | 10th |
| --- | --- | --- | --- | --- | --- | --- | --- | --- | --- | --- |
| Baseline measurement | √ |  |  |  |  |  |  |  |  |  |
| Online teacher workshops |  | √ |  |  |  | √ |  |  |  |  |
| Face-to-face teacher workshops |  |  |  | √ |  |  |  | √ |  |  |
| Dissemination of teacher booklet, parent booklet, FMS poster, simple sports equipment |  | √ |  |  |  |  |  |  |  |  |
| Coach demonstration at each school |  |  | √ |  |  |  |  |  |  |  |
| Class observation at each school |  |  |  | √ |  |  |  |  |  |  |
| Use of mobile application |  | √ | √ | √ | √ | √ | √ | √ | √ |  |
| Parent-child physical activity session |  |  |  |  |  |  | √ |  |  |  |
| Post-test |  |  |  |  |  |  |  |  |  | √ |

Topics of the teacher workshops:

- WHO 24-hour movement guidelines
- Fundamental movement skills (FMS) introduction
- FMS blended-in games
- Pedagogical theories and teaching strategies

Description of teaching materials:

- All teaching materials were delivered to each preschool after the baseline measurement and before the intervention
- The FMS poster consists of step-by-step diagram of each fundamental movement skills
- The teacher booklet involves detailed, step-by-step criteria of FMS based on TGMD-3. Examples of games blended with FMS are also provided, which can be achieved via the simple sports equipment
- The easy sports equipment consists of a ball, two bean-bag, a stick and a handkerchief.

Pictures of FMS poster (Figure a.), teacher booklet (Figure b.), simple sports equipment (Figure c.) and parent booklet (Figure d.)


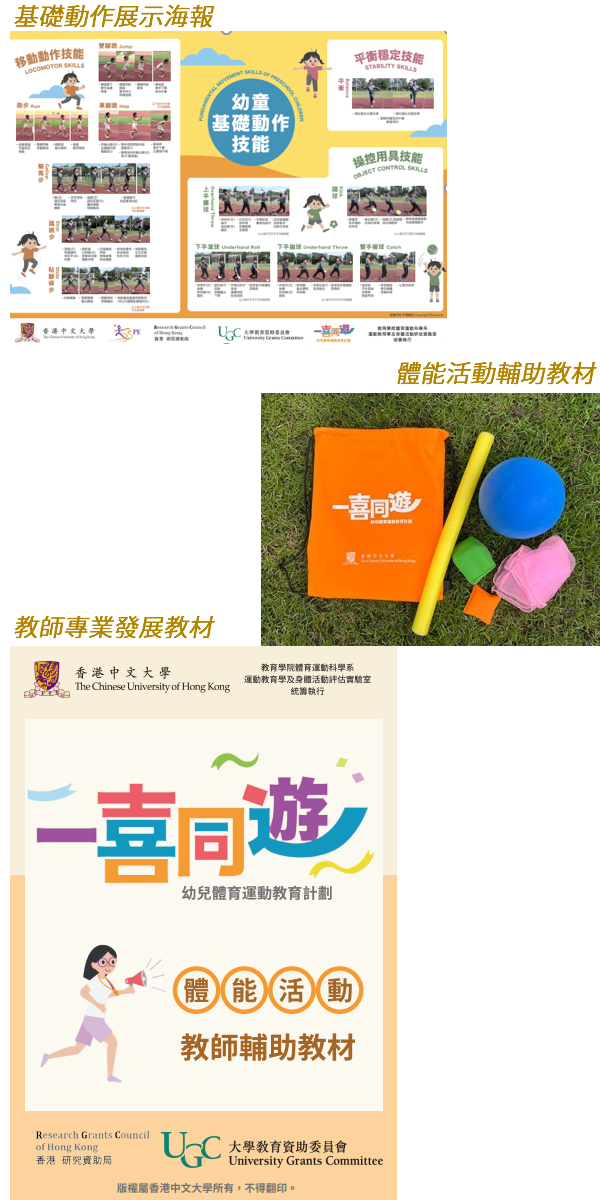

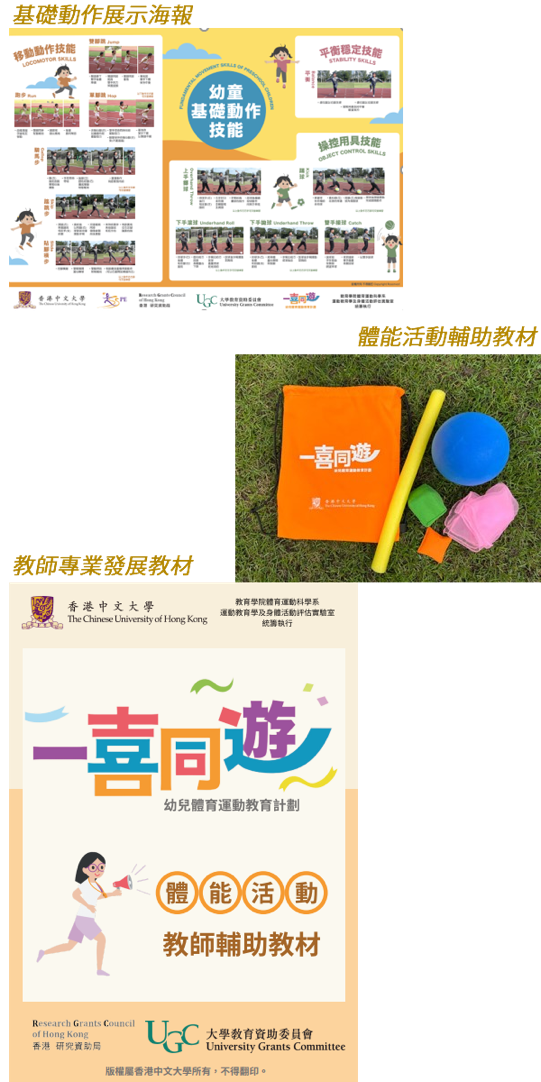


1. b.


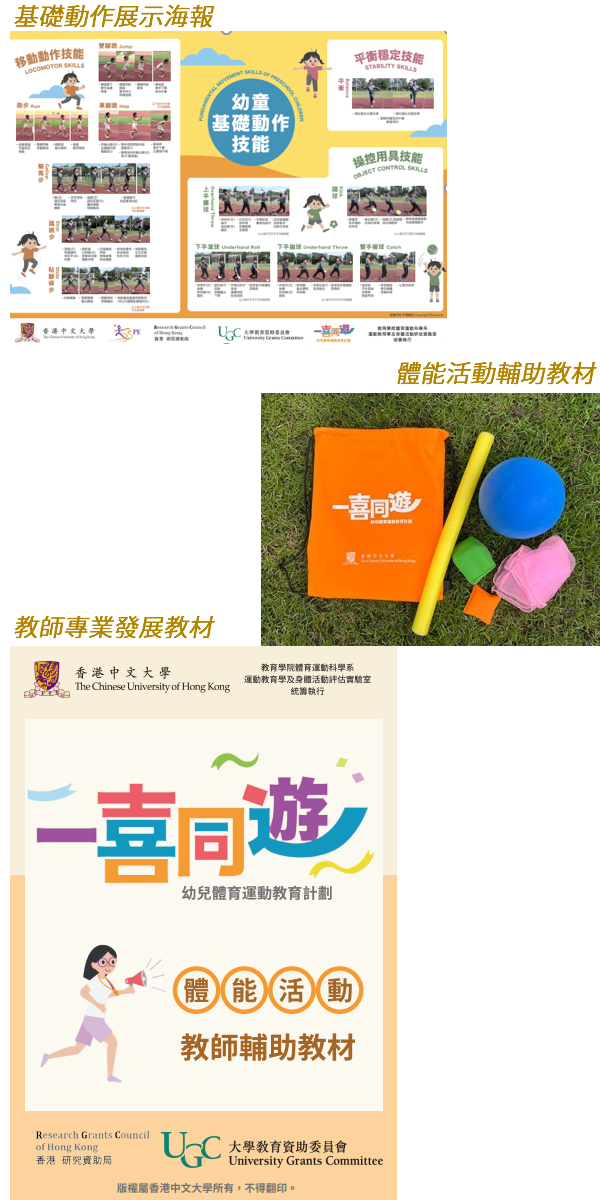

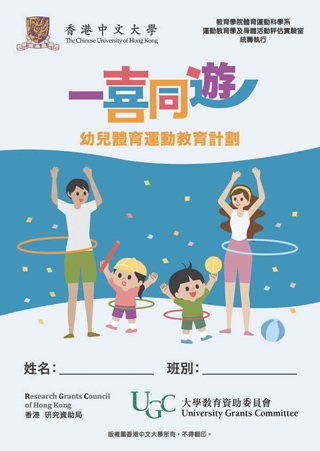


c. d.

Figures
